# Assessing mercury and lead pollution in the Ankobra estuary due to artisanal mining activities: Implications for water quality and aquatic life

**DOI:** 10.1371/journal.pone.0325909

**Published:** 2025-06-10

**Authors:** Mary Opeyemi Adebote, Joseph Aggrey-Fynn, Paul Kojo Mensah

**Affiliations:** 1 Department of Fish and Wildlife Conservation, Virginia Tech. 310 West Campus Dr., Blacksburg, Virginia, United States of America; 2 Centre for Coastal Management, Africa Centre of Excellence in Coastal Resilience (ACECoR), University of Cape Coast, Cape Coast, Ghana; 3 Department of Fisheries and Aquatic Sciences, School of Biological Sciences, College of Agriculture and Natural Sciences, University of Cape of Coast, Cape Coast, Ghana; Makerere University College of Natural Sciences, UGANDA

## Abstract

Artisanal and small-scale gold mining, “Galamsey” as it is called in Ghana, within the Ankobra estuary has impacted the water quality, fish health and other aquatic organisms. This paper presents a study investigating the concentrations of mercury and lead in water, sediments, gills and liver of *Chrysichthys nigrodigitatus* of the Ankobra estuary. Bimonthly sampling between December 2020 and June 2021 was conducted at nine locations along the estuary. Physicochemical parameters such as temperature, dissolved oxygen, pH and turbidity were determined in situ using the EUTECH Multi-Parameter probe. A total of 36 water samples, 36 sediment samples and 120 fish samples were collected. Mercury (Hg) and lead (Pb) concentrations in the samples were measured using Atomic Absorption Spectrophotometry (AAS), employing appropriate techniques for each metal. Results showed that the physicochemical parameters of water, except for turbidity, were within the acceptable limits for aquatic life, based on the United States Environmental Protection Agency water quality criteria. Mercury and lead concentrations in water and fish samples exceeded United States Environmental Protection Agency guideline thresholds. In sediments, mercury levels surpassed both average shale values and ecological risk thresholds (ERM = 0.71 mg/kg), indicating high ecological risk, while lead remained below the ERL (46.7 mg/kg), suggesting low risk. Mercury and lead concentration in fish, water and sediments differ significantly (P < 0.05) across locations and between matrices (water, sediment, and fish organs). High bioaccumulation factors (BAFs) were recorded for both metals in fish. The BAF of mercury in fish-to-water samples was greater than in fish-to-sediment samples. In contrast, the bioaccumulation factor of lead was greater than in sediments compared to water. The study revealed that Ankobra estuary is polluted with mercury and lead and all necessary regulations should be enforced on the activities of artisanal miners to curb this menace.

## Introduction

Toxic metals constitute a core group of aquatic pollutants due to their bio-accumulative and non-biodegradable properties. Activities such as mining, smelting, agriculture, domestic waste, sewage, and industrial discharges pollute the surrounding environment [1]. Artisanal and small-scale gold mining (ASGM) involves the informal extraction, and processing of alluvial gold in streams, estuaries and rivers informally with the use of basic tools and equipment. It is currently practiced in Central Asia, Latin America, Southeast and Africa, carried out by local communities with limited alternative financial opportunities, particularly in developing countries [2]. In Ghana, for example, gold constitutes two-thirds of production in the small-scale mining sector, with alluvial gold mining and mercury amalgamation being particularly common activities [2,3]. Similarly, in the Philippines, 90% of small-scale mining is dedicated to gold extraction, highlighting the sector’s significance in the country’s economy [2]. The Illegal small-scale gold mining activities in Ghana also known as “galamsey”, along with agricultural practices and indiscriminate waste disposal all impact the estuarine ecosystems. Galamsey results in the incessant release of untreated liquid waste from the washing of alluvial gold, and it is a key factor of pollution in the Ankobra estuary [4]. These activities are detrimental to the environment, especially the aquatic ecosystem, and the most hazardous pollutants from mining are toxic metals such as mercury and lead, amongst others [5]. The Ankobra estuary located in Ghana is associated with illegal small-scale gold mining activities which have become a national issue. Several studies have been conducted on illegal small-scale mining and its impact on biota within Ankobra estuary [6–9] however, little is known about its impact on aquatic species particularly, *Chrysichthys nigrodigitatus* in Ankobra Estuary.

Toxic metals in the aquatic environment can bioaccumulate and biomagnify in biota such as plankton, invertebrates and fish [9]. Fish are effective indicators for environmental biomonitoring due to their ability to bioaccumulate toxic metals, which then become biomagnified within the food chain [10]. The physiological processes of fish enable them to accumulate metals in significant concentrations, particularly in the liver and gills, which serve as primary sites of metal accumulation, where they can store metals for longer periods [11,12]. Lead and mercury accumulate in fish gills and liver and cause severe injury to the organs such as epithelial lifting and necrosis [13]. The metals have the potential to be neurotoxic, nephrotoxic, and carcinogenic [14,15].

*Chrysichthys nigrodigitatus,* the bagrid catfish, is one of the most important African commercial fishes with high economic value. It is one of the dominant fish caught in the Ankobra estuary and serves as a main source of protein for the communities surrounding the estuary [16]. This fish species is a benthic omnivore that occupies the secondary consumer position in the food pyramid [17]. Therefore, this study aims to evaluate the physiochemical parameters of the Ankobra estuary and investigate the mercury and lead concentration in water, sediments and *C. nigrodigitatus* samples from the estuary. Again, the study determined the bioaccumulation factor in the gills and liver of *C. nigrodigitatus* from the Ankobra estuary.

## Materials and methods

**Fig 1:**
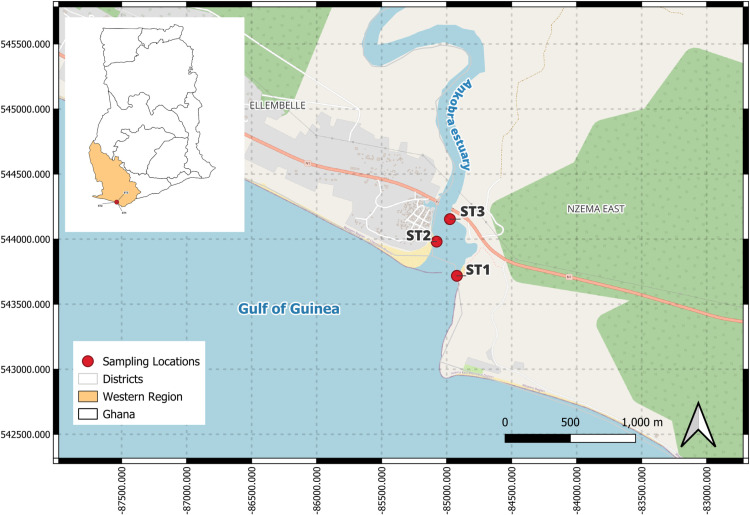
Map of study site showing the sampling stations in Ankobra estuary. Map created in QGIS using data from OpenStreetMap contributors, public domain spatial datasets, and shapefiles developed in ArcGIS Pro.

The Ankobra estuary is one of the south-western basins of Ghana. It is located within latitudes 4°52′–6°27′N, and longitudes 1°42′–2°33′W (Fig 1). It is bounded to the East, West and South by the Pra Basin, Tano Basin, and the Gulf of Guinea respectively [18]. The Basin originates in the hills north of Dare Basin and flows for roughly 260 kilometers largely before entering in the south into the Gulf of Guinea at Asanta, just west of Axim [19]. Ankobra Estuary has rich biodiversity characterized by terrestrial forests, swamp, bamboo, and mangrove forests. The West African dwarf crocodile (*Osteolaemus tetraspis*) and the cassava croaker (*Pseudotholithus elongates*) are among the fauna species of worldwide conservation concern [16]. Among the 27 estuarine fish species identified, the most important to the fishers are the cassava croaker (*Pseudotolithus spp.*), the Nile tilapia (*Oreochromis niloticus*), and the bagrid catfish (*Chrysichthys nigrodigitatus*) [16]. The major activity in this area is artisanal gold mining and this accounts for about 10 percent of total Ghana gold production [5]. Fishing is done all year round in the Estuary but peaks in the rainy season when fish is relatively abundant.

No specific permits were required for field site access, as the study was conducted in publicly accessible areas where research activities did not involve restricted zones or protected species. However, local authorities were informed about the study. Bi-monthly samplings from nine locations within the Ankobra estuary were undertaken from December 2020 to June 2021, covering both dry and wet seasons typical of coastal Ghana. December and February represent dry season months, while April and June fall within the wet season. Water and sediment sampling were taken at three sampling stations (Fig 1), with three different spots sampled at each station. Stations 1, 2 and 3 are located within latitudes 4°53′60”- 4°56”40’′N and longitudes 2°16′40”- 2°13′30"W. Physicochemical parameters of water including temperatures, pH, salinity and dissolved oxygen were measured *in situ* using an EUTECH Multi-Parameter probe (PCD650). The turbidity and depth were measured using the turbidimeter and sonar gun respectively. Water sample collections at each sampling station every other month from December 2020 to June 2021 were carried out for toxic metals analysis. Sampled water was stored in 500-mL capped plastic bottles at each sampling point [20]. The sample bottles were rinsed with the site water, and then water was collected in the container 15 cm below the water surface. Sampled water was fixed with concentrated nitric acid to ensure metal stabilization [20]. The sampling bottles were labeled appropriately, kept in an ice chest and transported to the Department of Fisheries and Aquatic Sciences laboratory for analysis.

Sediment samples were collected with an Ekman grab sampler for heavy metals analysis directly from the bottom of the water into zip-lock plastic bags at each sampling location. A total of 36 sediment samples were collected at each sampling point throughout the study period. Ekman grab was deployed from a boat into the bottom of the estuary at different stations to retrieve sediment samples. Sediment samples were air-dried for three weeks to complete dryness. They were then ground to powder with porcelain mortar and pestle and sieved through 63- and 125-micron mesh to obtain smooth and fine particles. Fine particles are preferred because they are closely packed and contain high levels of metal concentration. About 100g of sediment samples were weighed and stored in smaller zip-lock bags, kept in a cool dry place until toxic metal analysis. Concentrations of mercury (Hg) and lead (Pb) in sediments were analyzed and averaged to assess overall contamination levels. These mean concentrations were compared against average shale values [21] for enrichment assessment and against Effects Range Low (ERL) and Effects Range Median (ERM) guidelines [22] for ecological risk evaluation.

Fish samples were obtained from local fishermen. This study did not involve the handling or interaction with live animals. The fish species used for this research were deceased at the time they reached the shore. The fish were captured by local fishers using gill nets as part of routine fishing activities, and it is understood that their mortality likely resulted from stress or asphyxiation during the capture process. As such, this study does not require ethical clearance or approval from animal care and use committees. However, we affirm that all data collection and research practices were conducted responsibly and in accordance with applicable ethical standards. The fish samples were transported in an ice chest to the Department of Fisheries and Aquatic Sciences, University of Cape Coast. Samples were washed and prepared for morphometrics.

A total of 36 water samples, 36 sediment samples and 120 fish samples were collected throughout the study. The total length (TL) and body weight (BW) of each fish specimen were measured using a measuring board and a digital scale. Fish specimens (gills and livers) were removed by dissecting the fish samples. They were stored in well labeled aluminum foil and kept frozen at -4^o^ C for toxic metals analysis. The concentration of toxic metals was measured using a PINAAcle 900T Perkin Elmer Atomic Absorption Spectrophotometer [23]. Mercury (Hg) concentrations were determined in all samples using a Perkin Elmer Flow Injection Analysis System – Atomic Absorption Spectrophotometer (FIAS-AAS) with the Cold Vapor Technique. Lead (Pb) concentrations were measured using a Perkin Elmer Flame Atomic Absorption Spectrophotometer (FAAS) with an air-acetylene flame. These instruments and techniques were selected to ensure precise and reliable quantification of Hg and Pb in the analyzed samples [24].

In this study, rigorous quality control measures were implemented to ensure the analytical reliability of AAS data. Quantification of toxic metals was performed using external calibration curves prepared with certified reference standards. These calibration curves were generated with at least five standard concentrations, ensuring a high correlation coefficient (R² > 0.995) for accuracy. Periodic recalibration was conducted after every 10 sample measurements to maintain consistency and mitigate instrumental drift. The entire analytical process followed established protocols as recommended by the American Public Health Association [20].

To validate the accuracy of the analytical process, certified reference materials (SRMs) were incorporated into each digestion and analysis batch. These included SRM 1947 (Lake Michigan Fish Tissue, NIST, USA), DOLT-3 (Dogfish Liver, National Research Council, Canada), and ISE Sample 999 (Moist Clay, WEPAL, Ivory Coast). The measured concentrations of metals in these reference materials closely matched the certified values, with recovery percentages ranging from 97.0% to 99.6%, confirming method reliability (S1 Table). Additionally, triplicate analysis was performed to ensure precision and blank samples were included to account for potential contamination. These quality control procedures collectively guarantee the robustness of the reported data [23].

The bioaccumulation factor was calculated to evaluate the accumulation of toxic metals in fish. It indicates the extent to which fish are exposed to and accumulate these metals, which can have significant ecological and health implications. The ratio of mercury and lead concentrations in fish organs and its dissipated concentration in the environment (water and sediments) was calculated according to [25,26], as follows:

Bioaccumulation factor for water samples.


$$BAF=\;\frac{Pollutant\;(toxic\;metals\;concn)in\;fish\;organs\;(mg/L)}{Pollutant\;(toxic\;metals\;concn)in\;water\;(mg/L)\;}$$

Sediment bioaccumulation factor


$$BAF=\;\frac{Pollutant\;(toxic\;metals\;concn)in\;fish\;organs\;(mg/L)}{Pollutant\;(toxic\;metals\;concn)in\;sediment\;(mg/L)\;}$$

The concentration of mercury (Hg) and lead (Pb) values of the fish specimens and sediment samples was in mg/kg and mg/kg is equivalent to mg/L.

### Statistical analysis

Pearson’s bivariate correlation analysis was first performed to assess the relationships among physicochemical parameters of water and concentrations of mercury (Hg) and lead (Pb) in water, sediment and fish specimens. This step helped identify significant associations among variables and determine the suitability of the dataset for Principal Component Analysis (PCA). PCA was subsequently used to assess the relationships among physicochemical parameters and mercury and lead concentrations in water, sediment, and fish organs. Variables were scaled and centered prior to analysis, and a variable correlation circle was used to visualize the relative contribution and direction of each variable to the principal components. Physicochemical parameters of sediments (e.g., pH, grain size, organic matter) were not measured in this study, as the primary focus was to assess mercury and lead contamination levels in sediments relative to ecological risk guidelines and geochemical background values. Prior to performing ANOVA, the normality of the data was assessed using the Shapiro-Wilk test, and the homogeneity of variances was checked using Levene’s test. One-way ANOVA was used in determining the difference between mercury and lead concentrations in the gills and liver of *C. nigrodigitatu*s, with a post-hoc test to further investigate pairwise differences. A two-way ANOVA was applied to assess the effects of sampling station and month on the concentration of mercury and lead in water and sediment. However, due to insufficient replicates across seasons, we did not include season as a factor in the Two-way ANOVA, as this would have compromised the statistical robustness of the analysis. All statistical analyses were performed at a significance level of p < 0.05 using Microsoft Excel 2021 (Microsoft Corporation, Redmond, Washington, USA) and R software version 4.2.2 (developed by the R Core Team, R Foundation for Statistical Computing, Vienna, Austria) [27,28].

## Results

### Physicochemical parameters of water

Salinity was generally low in station 2 and station 3 and the highest values of salinity were recorded in station 1 (Table 1). The temperatures in all stations were between 27°C to 32°C. Dissolved oxygen recorded showed temporal fluctuations in all the stations, with values varying throughout the months. There was no significant temperature or dissolved oxygen difference between the stations (P > 0.05; Table 1). Mean pH values recorded were 7.45–8.26, 7.52–7.82, 6.64–7.44, 6.99–7.16 for December 2020, February 2021, April 2021 and June 2021 respectively. There were fluctuations in the turbidity value throughout the study period. Station 1 had lower turbidity compared to other stations in all the months (Table 1) and there was a statistically significant difference between stations (P < 0.05; Table 1 and 2). Generally, the stations varied in depth throughout the study period. ANOVA showed a statistically significant difference in the stations (P < 0.05) and no statistically significant difference in the month (P > 0.05). Station 3 had the highest depth. Tukey’s post hoc test indicated significant differences between the stations in all physicochemical parameters (Table 2).

**Table 1:** Summary of physicochemical parameters (Mean, Min, Max, StDev, and P-value) across sampling stations in the Ankobra estuary.

Parameters	Station	Mean	Min	Max	StDev	P-value
**Temperature (** ^**o**^ **C)**	St 1	29.31 ± 0.285	27.7	30.3	0.99	0.951
	St 2	29.17 ± 0.338	27.2	30.2	1.17	
	St 3	29.28 ± 0.386	27.3	31.8	1.34	
**Salinity (ppt)**	St 1	8.63 ± 2.59	0.21	23.57	8.97	0.001
	St 2	1.33 ± 0.561	0.07	5.00	1.94	
	St 3	0.15 ± 1.801	0.07	0.50	0.13	
**Dissolved oxygen (mg/L)**	St 1	6.339 ± 0.231	5.04	7.52	0.79	0.109
	St 2	5.708 ± 0.110	5.02	6.29	0.38	
	St 3	6.009 ± 0.247	5.01	7.51	0.86	
**pH**	St 1	7.398 ± 0.0533	7.05	7.64	0.18	0.870
	St 2	7.436 ± 0.195	6.41	8.57	0.67	
	St 3	7.512 ± 0.178	6.77	8.72	0.62	
**Turbidity (NTU)**	St 1	128.5 ± 34.1	17.6	312.2	118.2	0.000
	St 2	382.8 ± 11.3	328.6	437.4	39.1	
	St 3	405.1 ± 15.0	338.6	468.8	51.9	
**Depth (m)**	St 1	0.7095 ± 0.046	0.488	1.006	0.16	0.000
	St 2	2.899 ± 0.307	0.914	4.416	1.06	
	St 3	3.161 ± 0.207	2.042	4.420	0.71	

**Table 2:** Tukey’s Post Hoc test results of the physico-chemical parameters (Mean ± Standard Error).

Parameters	Station 1	Station 2	Station 3
**Temperature (oC)**	29.31 ± 0.29^a^	29.17 ± 0.34^a^	29.28 ± 0.39^a^
**Salinity (ppt)**	8.63 ± 2.59^a^	1.33 ± 0.56^b^	0.15 ± 0.04^c^
**DO (mg/L)**	6.34 ± 0.23^a^	5.71 ± 0.11^a^	6.0 ± 0.25^a^
**pH**	7.4 ± 0.05^a^	7.4 ± 0.19^a^	7.5 ± 0.18^a^
**Turbidity (NTU)**	128.5 ± 34.1^a^	382.8 ± 11.3^a^	405.1 ± 15.0^b^
**Depth**	0.71 ± 0.05^a^	2.89 ± 0.31^a^	3.2 ± 0.21^b^

Superscripts “a” and “b” show where the differences between the stations lie

### Toxic metals concentrations

**Fig 2:**
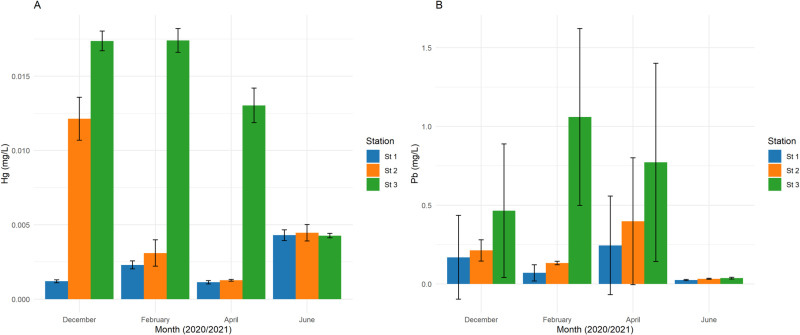
Mean concentration (±S.E.) of mercury and lead in water samples from the Ankobra estuary. The bar plot shows the mean concentrations of mercury (a) and lead (b) (mg/L) in water samples across different months for three sampling stations in the Ankobra estuary. Bars represent average concentrations with standard deviation error bars. Tukey’s post hoc test indicates significant differences among stations, with letters ‘a’, ‘b’, and ‘c’ for mercury and ‘a’ and ‘b’ for lead.

Metal concentrations varied throughout the months. Although not statistically tested, seasonal context informed interpretation (dry: Dec – Feb; wet: Apr – Jun). Mercury concentrations show variations in the water column (stations 1, 2, and 3) for all sampling months (Fig 2). Mercury levels in station 3 showed a temporary decrease over time, with the highest concentrations recorded in February 2021 (station 3) and December 2020 (station 2). The concentrations of lead were consistently higher in station 3 compared to other stations (Fig 2). A two-way ANOVA revealed significant spatial and temporal differences in mercury and lead levels (p < 0.05, S2 Table). The interaction in mercury levels between the month and stations was significant (p < 0.05) while in lead interaction was not significant (p = 0.192, S3 Table).

**Fig 3:**
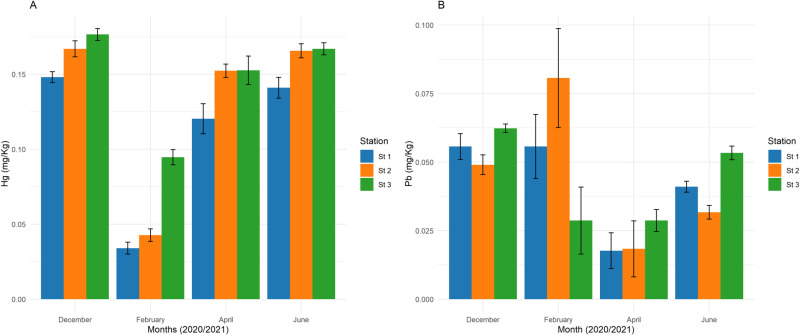
Mean concentration (±S.E.) of mercury and lead in sediment samples from the Ankobra estuary. The bar plot displays the mean concentrations of mercury (a) and lead (b) (mg/Kg) in sediment samples across different months for three sampling stations in the Ankobra estuary. Bars represent average concentrations with standard deviation error bars. Tukey’s post hoc test indicates significant differences among stations, with letters ‘a’, ‘b’, and ‘c’ for mercury and no difference observed in lead across stations.

Mercury concentrations in sediment samples show variations across the stations and months (Fig 3). Hg levels were lower in February 2021, ranging from 0.03 to 0.99 mg/kg. Statistically significant differences were found between stations (p < 0.05; S4 Table). Lead concentrations in sediment samples were generally low in April 2021, with the highest value recorded at station 2 in February 2021 (9.0 mg/kg) (Fig 3). A two-way ANOVA showed significant temporal differences (p > 0.05; S5 Table) but no significant spatial differences (p < 0.05), and the interaction between spatial and temporal factors was significant (p < 0.05; S5 Table). Mercury concentrations exceeded ecological risk benchmarks (ERM), indicating high risk, while lead remained below the ERL threshold, suggesting low risk (Table 3).

**Table 3:** Comparison of mean sediment mercury and lead concentrations with average shale values and sediment quality guidelines (ERL/ERM) for ecological risk assessment.

Metal	Measured Mean (mg/kg)	Average Shale (mg/kg)	ERL (mg/kg)	ERM (mg/kg)	Risk Interpretation
**Mercury (Hg)**	13.02	0.4	0.15	0.71	Exceeds ERM – High Risk
**Lead (Pb)**	5.66	20	46.7	218	Below ERL – Low Risk

**Fig 4:**
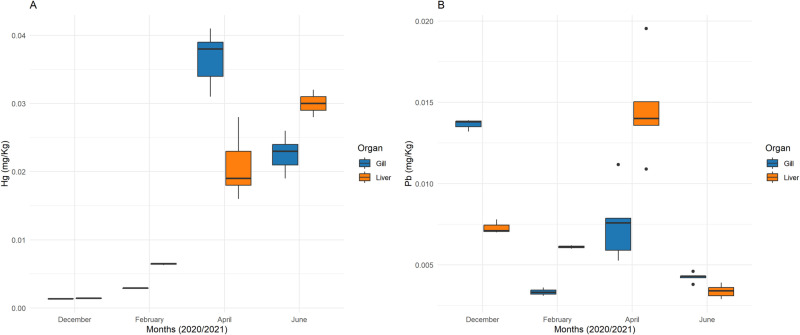
Mean concentration (±S.E.) of mercury and lead in *C. nigrodigitatus* from Ankobra estuary. The box plot shows the mean concentrations of mercury (a) and lead (b) (mg/Kg) in fish samples from the Ankobra estuary across the sampling months. Bars represent average concentrations with standard deviation error bars.

The mean mercury (Hg) concentration in gills was highest in April 2021 (3.9 mg/kg) (Fig 4). There was no significant difference between the two organs, but a significant difference was observed across the months (p < 0.05; S6 Table). There were fluctuations in lead (Pb) levels in the organs of *Chrysichthys nigrodigitatus*, with the highest levels also in April 2021 (Fig 4). While no significant difference was found between organs (p = 0.036; S7 Table), both the monthly variations and the interaction between month and organs were statistically significant (p < 0.05).

### Fish morphometric characteristics and relationship with metal concentration

The morphometric characteristics of *Chrysichthys nigrodigitatus* across the study period are presented in Table 4. The total length of fish samples ranged from 33.3 ± 1.4 cm in February 2021 to 37.1 ± 0.7 cm in April 2021, with the highest mean length observed in April. Body weights ranged from 331.2 ± 46.4 g in February to 385.4 ± 19.3 g in April. Variation in length and weight was observed across months, but no statistically significant difference was found (p > 0.05). Pearson’s correlation analysis using monthly mean values showed moderate to strong positive associations between fish size and metal concentrations, though none were statistically significant (p > 0.05), likely due to small sample size. For total length, the strongest correlations were observed with lead concentration in liver (r = 0.91, p = 0.087) and mercury in gill tissues (r = 0.86, p = 0.140). Similar trends were found for body weight, with correlations of r = 0.75 (p = 0.252) for Pb in liver and r = 0.71 (p = 0.290; S8 Table) for Hg in gill. While these results are not statistically significant, they suggest potential size-related bioaccumulation patterns, particularly for lead in liver and mercury in gills. Further investigation with larger sample sizes is warranted.

**Table 4:** Monthly variation in total length and body weight of *Chrysichthys nigrodigitatus* sampled from the Ankobra estuary.

Month	Mean Total Length (cm) ± SD	Length Variance	Length CoefVar (%)	Mean Body Weight (g) ± SD	Weight Variance	Weight CoefVar (%)	P-value
**Dec/20**	33.66 ± 1.71	58.49	22.72	364.1 ± 47.1	44404.7	57.87	0.218
**Feb/21**	33.33 ± 1.43	41.14	19.25	331.2 ± 46.4	43099.3	62.68	0.218
**Apr/21**	37.05 ± 0.66	8.813	8.01	385.4 ± 19.3	7419.5	22.35	0.218
**Jun/21**	33.81 ± 1.60	51.3	21.19	355.1 ± 42.6	36253.4	53.62	0.218

### Pearson correlation analysis

**Fig 5:**
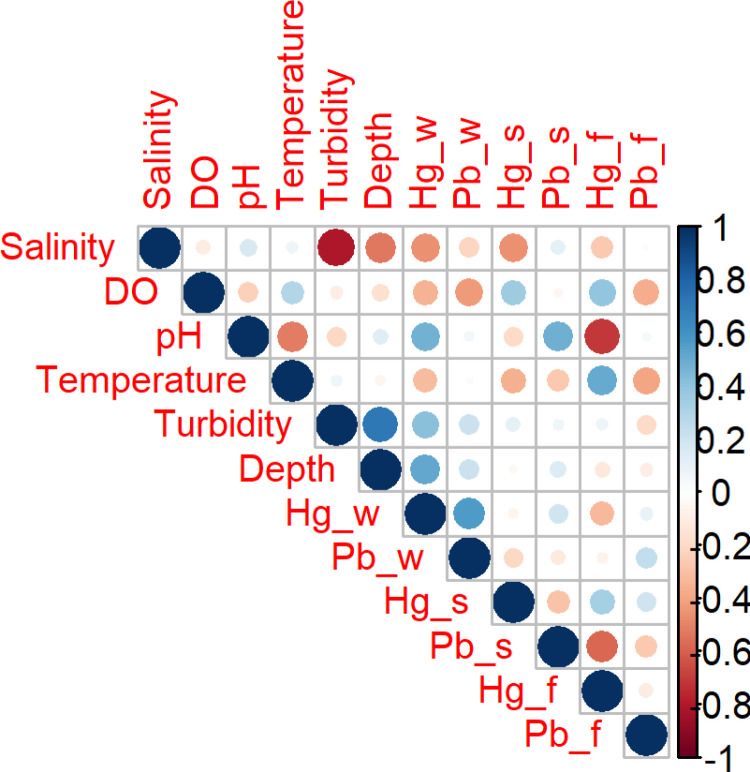
Pearson correlation matrix showing relationships between physicochemical parameters and toxic metal concentrations in the Ankobra estuary. Circle size and color indicate the strength and direction of correlations (blue: positive, red: negative), with larger circles representing stronger associations.

Pearson correlation analysis was conducted to examine relationships among physicochemical parameters and concentrations of mercury (Hg) and lead (Pb) in water, sediment, and fish organs. Strong positive correlations were observed between turbidity, depth, and metal concentrations in water (Hg_w, Pb_w), while salinity showed inverse associations with these variables (Fig 5). Additionally, dissolved oxygen (DO) exhibited a positive correlation with mercury in fish tissues (Hg_f). These observed relationships provided a basis for multivariate analysis using Principal Component Analysis (PCA) to further elucidate underlying environmental gradients.

### Principal component analysis

**Fig 6:**
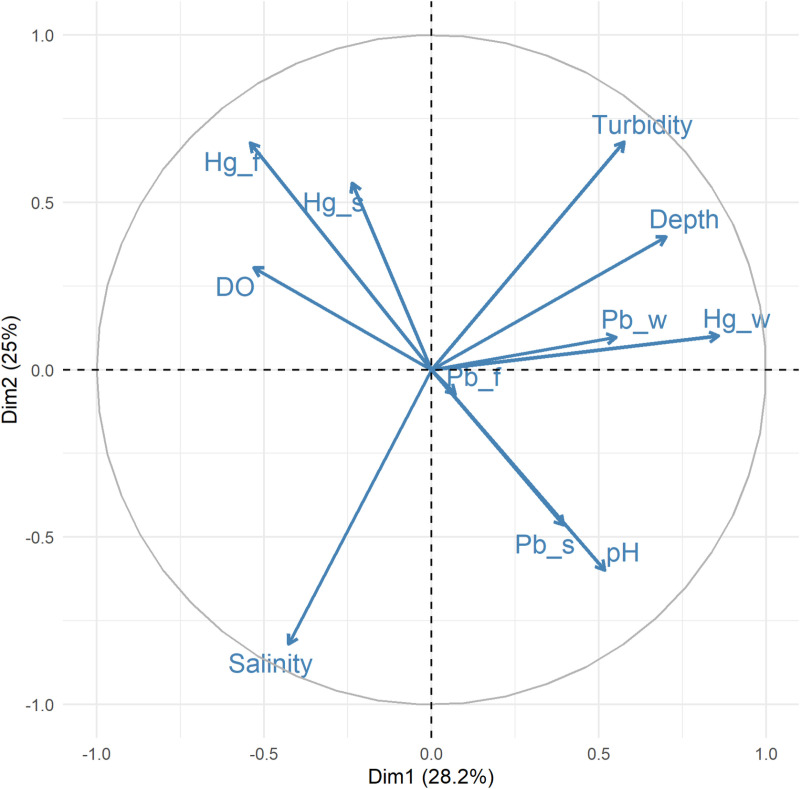
Principal Component Analysis (PCA) biplot showing the relationships among physicochemical parameters of water and toxic metals in water, sediment, and fish tissues. Variables are projected along Principal Component 1 (PC1: 28.2% variance) and Principal Component 2 (PC2: 25.0% variance), illustrating their correlations and contributions to observed environmental gradients.

Principal Component Analysis (PCA) was used to explore patterns among physicochemical parameters and concentrations of mercury (Hg) and lead (Pb) in water, sediment and fish organs. Significant positive correlations were observed between turbidity and lead in water (Pb_w), as well as between dissolved oxygen (DO) and mercury in fish tissues (Hg_f) (Fig 6). These relationships, along with other observed associations among physicochemical parameters and metal concentrations across environmental compartments, supported the subsequent use of PCA to uncover underlying multivariate patterns. The first two principal components explained 28.2% (PC1) and 25.0% (PC2) of the total variance, respectively, accounting for a cumulative variance of 53.2% (Fig 6). The PCA variable plot revealed distinct associations among environmental variables. Waterborne metal concentrations (Hg_w and Pb_w) were strongly aligned with depth and turbidity, suggesting co-variation influenced by mining-related factors. In contrast, fish mercury concentrations (Hg_f) and dissolved oxygen (DO) loaded negatively along PC1, indicating a possible inverse relationship with metal enrichment in the water column. Sediment-bound lead (Pb_s) and pH showed similar loading directions along PC2, suggesting that pH may influence sediment metal dynamics. Salinity loaded primarily along PC2, showing independence from the metal-related variables. These patterns suggest that both physicochemical gradients and anthropogenic inputs shape metal distribution in the Ankobra estuary. Full details of all statistical tests are available in the Supplementary Information (S9 Table).

### Bioaccumulation factor in *C. nigrodigitatus*

The bioaccumulation factor of toxic metals from water and sediments in the gills and liver of *C. nigrodigitatus* throughout the study period is shown in Table 5. The results showed that the bioaccumulation factor of mercury in fish organ specimens from water was greater than that from sediment. The bioaccumulation factor of lead in fish organ specimens showed a higher value from sediments than in water except for June 2021 (Table 5). Mercury levels in the liver of *C. nigrodigitatus* were above 0.002 mg/L United States Environmental Protection Agency maximum contaminant level goal in all the sampling months except for December 2020. Mercury levels in gills varied by month, with samples below the standard limit in December 2020 and February 2021 and above in April and June 2021. Lead concentrations in the gills and liver of *Chrysichthys nigrodigitatus* for the study period were above the recommended level of 0.3 mg/Kg set by the United States Environmental Protection Agency (US EPA) [29].

**Table 5:** Bioaccumulation Factor (BAF) of mercury and lead in gills and liver of *C. nigrodigitatus* from Ankobra estuary.

Month	Parameter	Hg	Pb
**December 2020**	Gills/Water	13.5	4.82
	Gills/Sediment	0.824	27.2
	Liver/Water	14.2	2.59
	Liver/Sediment	2.49	13.27
**February 2021**	Gills/Water	38.16	0.784
	Gills/Sediment	5.08	6.0
	Liver/Water	85.13	1.45
	Liver/Sediment	11.35	11.09
**April 2021**	Gills/Water	732	1.605
	Gills/Sediment	25.77	37.8
	Liver/Water	416	3.1
	Liver/Sediment	14.65	73
**June 2021**	Gills/Water	282.5	13.68
	Gills/Sediment	14.3	10.09
	Liver/Water	375	10.9
	Liver/Sediment	18.99	8.048

## Discussion

### Physicochemical parameters

The quality of water is defined by a set of general parameters, such as dissolved oxygen (DO), alkalinity, pH, hardness, conductivity, salinity, and turbidity [30]. In this study, salinity, temperature, dissolved oxygen, pH, turbidity and depth were the important parameters considered because of their relations with the release of toxic metals which showed levels of spatial and seasonal variations. The mean salinity ranges between 0.06 and 5.38 ppt recorded in this study (Table 1) a typical characteristic of an estuarine ecosystem [31,32] with a wide range of salinity. The high salinity in Station 1 could be attributed to its closeness to the sea with the influx of salty water, and the low level of salinity in Station 3 was recorded due to its proximity to the river. Temperatures recorded during the study were within the range of 27–32°C, with the hottest in February, 2021. The high salinity therefore could be related to the hot ambient temperature during that time of the year and minimal rainfall. The Ankobra estuary is generally characterized by consistently high temperatures all year round, with an average annual temperature of around 26°C, according to available meteorological data [33].

The dissolved oxygen (DO) levels in the Ankobra estuary were above the 5 mg/L stated as the threshold required to support aquatic life [34]. A study in Ghana by [32], recorded similar levels of dissolved oxygen in Domini and Amansumi Lagoon. The higher level of DO in June 2021 could be attributed to the major rainfall during the time period [35]. Generally, most aquatic organisms prefer a 6.5 - 9.0 pH range [30] and the mean pH level recorded for this study ranges from 7.1 - 8.3.

The mean turbidity levels recorded high values in the Ankobra estuary throughout the study period ranged from 20 - 464 NTU (Table 1). A similar study [36] showed high turbidity in the Pra estuary associated with mining activities, with levels exceeding 150 NTU for most periods of the year. Phytoplankton, algae development, sediment re-suspension from the bottom, urban runoff, and other factors can affect the turbidity of water bodies [37]. The high turbidity values recorded in Stations 2 and 3 compared to Station 1 (Table 1) may be a result of their proximity to the illegal mining (Galamsey) site upstream. These activities disturb the bottom of the Ankobra estuary leading to the suspension of silt particles in the water. The estuary is an important habitat for juvenile fishes of many species including *C. nigrodigitatus.* These juvenile fish may be affected by high turbid water causing gill deformation, weight loss and mortality [38].

Depth is also an important factor in aquatic ecosystems. The mean level of depth recorded in this study ranges from 0.6 to 3.9 m. The lower level recorded in Station 1 could be attributed to its nearness to the shore compared to the other stations (Table 1 and Fig 1). The first principal component of the physicochemical parameters of water, explaining the largest portion of the variance (28.2%), is heavily influenced by turbidity and depth. Both of these parameters are positively correlated, meaning they tend to increase together along this component. In contrast, salinity is negatively correlated with this component, indicating that as turbidity and depth increase, salinity tends to decrease. This suggests that areas with higher turbidity and depth tend to have lower salinity levels, which may reflect the influx of fresh water from the river and waste from Galamsey activities into the estuary [33].

The second principal component, which explained 25.0% of the variance, is characterized by a positive relationship with pH. This may indicate geochemical variation related to estuarine mixing or buffering capacity.The toxicity of toxic metals to aquatic life is affected by rising pH. Toxic metals precipitate and adsorb onto soil surfaces at higher pH levels. Metals are also more easily seeped into the water at lower pH [31]. Dissolved oxygen (DO), while not a major contributor to PC2, showed an inverse relationship with PC1 and certain metal variables in both the PCA and correlation matrix, suggesting that oxygen availability may be reduced in areas with elevated contamination and turbidity, potentially indicating zones of environmental stress.

### Toxic metal concentrations

The concentration of mercury in water from the Ankobra estuary was above 0.001 mg/L set by US Environmental Protection Agency (EPA) guidelines for the protection of aquatic life [39]. A similar study in the Pra river basin recorded high mercury (Hg) levels (> 0.03 mg/L) above the standard limit of the EPA [40]. A global literature review highlights that ASGM activities often lead to mercury contamination, adversely affecting ecosystems and posing health risks to miners and surrounding communities [41]. Artisanal mining in the Bozoum region of the Central African Republic has caused significant water contamination, with mercury concentrations in the Ouham River ranging from 4 to 26 µg/L, exceeding the standard limit by 26 times [41]. The level of mercury in sediments was higher than that of water, which is consistent with findings from other ASGM-impacted regions, where sediments act as long-term reservoirs for contaminants [42]. A comprehensive review by [41] highlights the environmental pollution and human health implications of ASGM, with mercury concentrations frequently exceeding safe thresholds in various African water bodies [43]. Likewise, [44] reported persistent mercury contamination in aquatic ecosystems linked to ASGM, emphasizing its bioaccumulative effects on fish and associated human health risks. Generally, the concentration of mercury in the gills and liver of *C. nigrodigitatus* was above (0.002 mg/Kg) US EPA maximum contaminant level goal [29]. The high values could be related to their tendency to accumulate more since they are at the top of the food pyramid (secondary consumers). A report from [40] showed higher concentrations of mercury in *C. nigrodigitatus* compared to other species in the study conducted in the Pra river basin. The elevated mercury levels may be attributed to the species’ trophic position as a secondary consumer, leading to increased bioaccumulation through the food chain. Similar trends were observed in a study where piscivorous fish in mining-impacted environments exhibited higher Hg concentrations than primary consumers [45].

Lead levels in water samples were above US EPA guidelines (0.0025 mg/L) for the protection of aquatic life [46]. The concentrations in the sediment were higher than in the water, which could be due to suspended toxic metals in the water column settling in the sediments and accumulating over time. Lead (Pb), being a particle-reactive element, preferentially binds to sediments, leading to long-term accumulation [47]. Studies on the Benya and Narkwa Lagoons in Ghana similarly reported greater concentration of metals in sediment than in water [48]. Research has confirmed that sedimentary lead levels in Ghanaian estuaries were influenced by anthropogenic activities, including ASGM [20]. Similarly, a review of artisanal mining in Africa underscores the environmental degradation caused by mining activities, including the release of toxic metals like mercury and lead into the environment, which can lead to soil and water contamination [49]. The high mercury concentrations in sediments, surpassing ecological risk thresholds (ERM), indicate severe contamination likely linked to artisanal gold mining activities. These observations mirror reports from [40], mercury accumulation in estuarine sediments subjected to small-scale mining inputs. Conversely, lead concentrations remained below ecological risk thresholds, suggesting minimal current impact, possibly due to lower lead inputs or effective sediment sequestration dynamics in the study area. Although commonly applied to assess sediment contamination, indices such as the Contamination Factor, Index of Geoaccumulation, and Potential Ecological Risk Index require site-specific background values to ensure reliability. In this study, locally validated baselines for the Ankobra estuary are unavailable, and using generalized global values (e.g., average shale) could misrepresent natural variability. Therefore, sediment contamination was evaluated through direct comparisons with established sediment quality guidelines (ERL/ERM) to maintain analytical rigor and avoid introducing uncertainty.

Lead levels in *Chrysichthys nigrodigitatus* gills and liver were higher than the United States Environmental Protection Agency’s maximum contaminant level target of 0 mg/L. The Agency for Toxic Substances and Disease Registry states that there is no safe level of lead concentrations [50]. This indicates lead levels in the fish of this study are exceedingly high, which could be detrimental to fish health and make them unfit for human consumption. Chronic exposure to lead through fish consumption has been associated with neurological disorders, kidney dysfunction, and developmental impairments [51]. These findings necessitate continuous monitoring of toxic metals in ASGM-impacted ecosystems to safeguard aquatic life and public health.

While this study provides critical insight into the concentrations and bioaccumulation of mercury and lead in the Ankobra estuary, it does not include a quantitative human health risk assessment due to the absence of key exposure-related data (e.g., population behavior patterns, ingestion/inhalation rates, body weights, or exposure frequency). Nonetheless, the observed levels of toxic metals in water, sediment, and biota suggest potential public health risks, particularly in communities dependent on the estuary for livelihood and food. In the context of artisanal and small-scale gold mining (ASGM), direct physical contact with contaminated water, sediment, and mercury-containing materials are often without protective clothing is common and may constitute a significant pathway of exposure. Recent studies in Ghana have reported that miners frequently engage in gold recovery processes that involve bare-hand handling of mercury and prolonged skin contact with polluted environments, leading to increased health risks [52,53]. We recommend that future studies incorporate detailed exposure assessments, including dermal, inhalation, and ingestion pathways, to comprehensively evaluate both cancer and non-cancer health risks associated with metal pollution in ASGM-affected ecosystems.

PCA results showed that PC1 (28.2%) captured the influence of turbidity, depth, and waterborne Hg and Pb concentrations, suggesting a shared pattern likely driven by artisanal mining activities and water movement. These parameters may reflect increased particulate loading and associated metal transport. PC2 (25.0%) highlighted a different axis dominated by pH and salinity, indicating the influence of estuarine mixing and geochemical processes.

Mercury in fish tissues (Hg_f) loaded inversely along PC1, opposite to turbidity and waterborne metals, suggesting a potential bioaccumulation response under conditions of elevated contamination and lower dissolved oxygen. Sediment-bound lead (Pb_s) aligned more closely with pH along PC2, indicating that geochemical conditions may influence metal retention and mobility in the sediment. The inverse relationship between DO and toxic metal concentrations further supports the role of oxygen stress in shaping contaminant dynamics.

Recent studies demonstrated that increased particulate matter may facilitate mercury (Hg) accumulation in aquatic environments by increasing the binding of Hg to suspended particulates, thereby promoting its transport and bioavailability [52]. For instance, research published in 2024 highlights that a significant portion of mercury in streams is associated with suspended particulate matter, which can lead to increased bioaccumulation in aquatic food webs [53]. Similarly, depth variations could influence the resuspension and mobility of toxic metals, influencing their distribution and potential ecological risks [54]. Our findings are consistent with previous research, which reported that turbidity and depth are significant drivers of Hg distribution in aquatic ecosystems [2]. While the PCA results suggest that physicochemical parameters do not strongly cluster with mercury and lead concentrations overall, the observed associations suggest that turbidity and depth may still influence metal distribution. Future studies could further explore this interaction using sediment analysis and more targeted seasonal assessments.

The gills and liver of *C. nigrodigitatus* from Ankobra estuary accumulated more mercury in water than from sediments, indicating a stronger assimilation of mercury via the water column. Conversely, lead was more readily absorbed from sediment throughout the study period, except for June 2021. These patterns likely reflect Galamsey activities around the estuary, as similar high mercury concentrations have been recorded near artisanal gold extraction sites [55]. Bioaccumulation factors (BAFs) for both metals exceeded the United States Environmental Protection Agency maximum contaminant level goal, indicating significant bioaccumulation and biomagnification [29]. Compared to other regions, *C. nigrodigitatus* in the Ankobra estuary showed higher mercury bioaccumulation than fish from mining-impacted systems like the Amazon River basin [56], while estuarine fish in Southeast Asia similarly exhibited elevated lead levels in mining and industrial areas [24]. These comparisons underscore the substantial influence of anthropogenic activities on metal accumulation in aquatic ecosystems.

Additionally, positive correlations were observed between fish size and metal concentrations, particularly between total length and lead in liver (r = 0.91) and mercury in gills (r = 0.86), suggesting potential size-related bioaccumulation patterns despite the small sample size (n = 4 months). This trend aligns with findings by [57], who reported significant relationships between fish length and mercury accumulation in mining-impacted freshwater fish. Larger individuals tend to occupy higher trophic levels and accumulate metals over longer exposure durations, highlighting the need to consider fish size and age in future ecological risk assessments. While this study did not quantify human health risk indices due to limited exposure data, the elevated metal concentrations in water, sediment, and fish tissues present potential risks to communities reliant on the estuary. Future studies should incorporate exposure assessment models, including dermal and dietary pathways, to comprehensively evaluate risks in ASGM-impacted ecosystems.

## Conclusion

This study evaluated mercury and lead contamination in the Ankobra estuary, highlighting significant mercury enrichment in sediments and biota, primarily linked to artisanal and small-scale gold mining (Galamsey) activities. Mercury concentrations exceeded ecological risk thresholds, posing a clear environmental concern, while lead levels remained below critical limits, indicating a lower immediate risk. The observed bioaccumulation patterns in fish tissues underscore the estuary’s ecological vulnerability and suggest early warning signs of potential human health impacts for communities dependent on estuarine resources [58]. Due to the lack of exposure-specific data, this study did not quantify human health risk indices; however, the findings establish a vital ecological baseline for continued monitoring. Future research should prioritize comprehensive risk assessments incorporating exposure pathways, particularly for mercury. These results reinforce the need for integrated management strategies, including stricter environmental regulations, community engagement, and sustainable livelihood interventions, to mitigate the environmental and socio-economic impacts of artisanal mining in the Ankobra estuary.

## Supporting information

S1 Table Certified values, measured concentrations, and recovery rates of mercury and lead in standard reference materials used for analytical quality control. (DOCX)

S2 Table Anova and Tukey results of mercury concentrations in water (mg/L). (DOCX)

S3 Table Anova and Tukey results of lead concentrations in water (mg/L). (DOCX)

S4 Table Anova and Tukey results of mercury concentrations in sediments (mg/Kg). (DOCX)

S5 Table Anova and Tukey results of lead concentrations in sediments (mg/Kg). (DOCX)

S6 Table Anova and Tukey results of mercury concentrations in fish organs (mg/Kg). (DOCX)

S7 Table Anova and Tukey results of lead concentrations in fish organs (mg/Kg). (DOCX)

S8 Table Correlation between fish morphometrics and metal concentrations. (DOCX)

S9 Table PCA loadings of physicochemical parameters and toxic metal concentrations on the first two principal components (PC1 and PC2) for the Ankobra estuary. (DOCX)
