# Supplementary material for: Assessing mercury and lead pollution in the Ankobra estuary due to artisanal mining activities: Implications for water quality and aquatic life
Source: PLoS One. 2025 Jun 10;20(6):e0325909. doi: 10.1371/journal.pone.0325909 (PMC12151438; doi:10.1371/journal.pone.0325909)
Supplement: S1 Table — (DOCX) [file pone.0325909.s001.docx]

**S1 Table: Certified values, measured concentrations, and recovery rates of mercury and lead in standard reference materials used for analytical quality control.**

**S1a Table:** Certified mass fraction (wet-mass basis) and measured concentration of Hg in SRM 1947 Lake Michigan Fish Tissue

| **Analyte** | **Certified value (mg/kg)** | **Measured value (mg/kg)** | **% Recoveries** |
| --- | --- | --- | --- |
| Hg | 0.254 ± 0.005 | 0.270 ± 0.008 | 99.6 |

**S1b Table:** Certified and measured concentration of Pb in DOLT-3 dogfish liver National Reference Council Canada

| **Analyte** | **Certified value (mg/kg)** | **Measured value (mg/kg)** | **% Recoveries** |
| --- | --- | --- | --- |
| Pb | 0.03 ± 0.005 | 0.045 ± 0.005 | 99.6 |

**S1c Table:** Certified and measured concentration of Pb and Hg in ISE sample 999 of moist clay from Liteta/Ivory Coast, WEPAL

| **Analyte** | **Certified value (mg/kg)** | **Measured value (mg/kg)** | **% Recoveries** |
| --- | --- | --- | --- |
| Pb | 4.710 ± 1.157 | 4.907 ± 0.235 | 98.7 |
| Hg | 0.0102 ± 0.00127 | 0.0122 ± 0.00118 | 97.2 |
