# Supplementary material for: Assessing mercury and lead pollution in the Ankobra estuary due to artisanal mining activities: Implications for water quality and aquatic life
Source: PLoS One. 2025 Jun 10;20(6):e0325909. doi: 10.1371/journal.pone.0325909 (PMC12151438; doi:10.1371/journal.pone.0325909)
Supplement: S2 Table — (DOCX) [file pone.0325909.s002.docx]

**S2 Table:** Anova and Tukey results of mercury concentrations in water (mg/L)

|  | **Df** | **Sum Sq** | **Mean Sq** | **F value** | **Pr(>F)** |
| --- | --- | --- | --- | --- | --- |
| **Station** | 2 | 0.0007431 | 0.0003716 | 760.4 | < 2e-16 *** |
| **Month** | 3 | 0.0001908 | 0.0000636 | 130.1 | 5.58e-15 *** |
| **Station:Month** | 6 | 0.0003785 | 0.0000631 | 129.1 | < 2e-16 *** |
| **Residuals** | 24 | 0.0000117 | 0.0000005 |  |  |

Tukey results

|  | **Diff** | **lwr** | **upr** | **p adj** |
| --- | --- | --- | --- | --- |
| **St 2-St 1** | 0.00301 | 0.00230 | 0.00372 | 0 |
| **St 3-St 1** | 0.01078 | 0.01007 | 0.01150 | 0 |
| **St 3-St 2** | 0.00777 | 0.00706 | 0.00849 | 0 |
