# Supplementary material for: Assessing mercury and lead pollution in the Ankobra estuary due to artisanal mining activities: Implications for water quality and aquatic life
Source: PLoS One. 2025 Jun 10;20(6):e0325909. doi: 10.1371/journal.pone.0325909 (PMC12151438; doi:10.1371/journal.pone.0325909)
Supplement: S3 Table — (DOCX) [file pone.0325909.s003.docx]

**S3 Table:** Anova and Tukey results of lead concentrations in water (mg/L)

|  | **Df** | **Sum Sq** | **Mean Sq** | **F value** | **Pr(>F)** |
| --- | --- | --- | --- | --- | --- |
| **Station** | 2 | 1.458 | 0.729 | 7.12 | 0.0037 ** |
| **Month** | 3 | 1.049 | 0.350 | 3.41 | 0.0336 * |
| **Station:Month** | 6 | 0.981 | 0.164 | 1.60 | 0.1915 |
| **Residuals** | 24 | 2.458 | 0.102 |  |  |

Tukey results

|  | **Diff** | **lwr** | **upr** | **p adj** |
| --- | --- | --- | --- | --- |
| **St 2-St 1** | 0.0668 | -0.2595 | 0.393 | 0.867 |
| **St 3-St 1** | 0.4564 | 0.1301 | 0.783 | 0.005 |
| **St 3-St 2** | 0.3896 | 0.0633 | 0.716 | 0.017 |
