# Supplementary material for: Assessing mercury and lead pollution in the Ankobra estuary due to artisanal mining activities: Implications for water quality and aquatic life
Source: PLoS One. 2025 Jun 10;20(6):e0325909. doi: 10.1371/journal.pone.0325909 (PMC12151438; doi:10.1371/journal.pone.0325909)
Supplement: S4 Table — (DOCX) [file pone.0325909.s004.docx]

**S4 Table:** Anova and Tukey results of mercury concentrations in sediments (mg/Kg)

|  | **Df** | **Sum Sq** | **Mean Sq** | **F value** | **Pr(>F)** |
| --- | --- | --- | --- | --- | --- |
| **Station** | 2 | 0.00821 | 0.004105 | 118.7 | 3.60e-13 *** |
| **Month** | 3 | 0.06641 | 0.022135 | 639.8 | < 2e-16 *** |
| **Station:Month** | 6 | 0.00286 | 0.000477 | 13.8 | 9.49e-07 *** |
| **Residuals** | 24 | 0.00083 | 0.000035 |  |  |

Tukey results

|  | **Diff** | **lwr** | **upr** | **padj** |
| --- | --- | --- | --- | --- |
| **St 2-St 1** | 0.02106667 | 0.01506981 | 0.02706353 | 0.0e+00 |
| **St 3-St 1** | 0.03686667 | 0.03086981 | 0.04286353 | 0.0e+00 |
| **St 3-St 2** | 0.01580000 | 0.00980314 | 0.02179686 | 2.4e-06 |
