# Supplementary material for: Assessing mercury and lead pollution in the Ankobra estuary due to artisanal mining activities: Implications for water quality and aquatic life
Source: PLoS One. 2025 Jun 10;20(6):e0325909. doi: 10.1371/journal.pone.0325909 (PMC12151438; doi:10.1371/journal.pone.0325909)
Supplement: S5 Table — (DOCX) [file pone.0325909.s005.docx]

**S5 Table:** Anova and Tukey results of lead concentrations in sediments (mg/Kg)

|  | **Df** | **Sum Sq** | **Mean Sq** | **F value** | **Pr(>F)** |
| --- | --- | --- | --- | --- | --- |
| **Station** | 2 | 0.000037 | 0.0000184 | 0.266 | 0.769 |
| **Month** | 3 | 0.006877 | 0.0022922 | 33.207 | 1.05e-08 *** |
| **Station:Month** | 6 | 0.005225 | 0.0008708 | 12.615 | 2.09e-06 *** |
| **Residuals** | 24 | 0.001657 | 0.0000690 |  |  |

Tukey results

|  | **Diff** | **lwr** | **upr** | **p adj** |
| --- | --- | --- | --- | --- |
| **St 2-St 1** | 0.002416667 | -0.006053750 | 0.010887084 | 0.7585417 |
| **St 3-St 1** | 0.000750000 | -0.007720417 | 0.009220417 | 0.9734361 |
| **St 3-St 2** | -0.001666667 | -0.010137084 | 0.006803750 | 0.8760668 |
