# Supplementary material for: Assessing mercury and lead pollution in the Ankobra estuary due to artisanal mining activities: Implications for water quality and aquatic life
Source: PLoS One. 2025 Jun 10;20(6):e0325909. doi: 10.1371/journal.pone.0325909 (PMC12151438; doi:10.1371/journal.pone.0325909)
Supplement: S6 Table — (DOCX) [file pone.0325909.s006.docx]

**S6 Table:** Anova and Tukey results of mercury concentrations in fish organs (mg/Kg)

|  | **Df** | **Sum Sq** | **Mean Sq** | **F value** | **Pr(>F)** |
| --- | --- | --- | --- | --- | --- |
| **Organ** | 1 | 0.000030 | 0.0000302 | 3.71 | 0.066 |
| **Month** | 3 | 0.004550 | 0.0015168 | 186.43 | < 2e-16 *** |
| **Organ: Month** | 3 | 0.000750 | 0.0002500 | 30.72 | 2.19e-08 *** |
| **Residuals** | 24 | 0.000195 | 0.0000081 |  |  |

Tukey results

|  | **Diff** | **lwr** | **upr** | **p adj** |
| --- | --- | --- | --- | --- |
| **Liver-Gill** | -0.0019425 | -0.004023907 | 0.0001389067 | 0.0660088 |
