# Supplementary material for: Assessing mercury and lead pollution in the Ankobra estuary due to artisanal mining activities: Implications for water quality and aquatic life
Source: PLoS One. 2025 Jun 10;20(6):e0325909. doi: 10.1371/journal.pone.0325909 (PMC12151438; doi:10.1371/journal.pone.0325909)
Supplement: S7 Table — (DOCX) [file pone.0325909.s007.docx]

**S7 Table:** Anova and Tukey results of lead concentrations in fish organs (mg/Kg)

|  | **Df** | **Sum Sq** | **Mean Sq** | **F value** | **Pr(>F)** |
| --- | --- | --- | --- | --- | --- |
| **Organ** | 1 | 0.0000129 | 1.287e-05 | 4.936 | 0.036 * |
| **Month** | 3 | 0.0003641 | 1.214e-04 | 46.544 | 3.71e-10 *** |
| **Organ: Month** | 3 | 0.0001852 | 6.173e-05 | 23.676 | 2.38e-07 *** |
| **Residuals** | 24 | 0.0000626 | 2.610e-06 |  |  |

Tukey results

|  | **Diff** | **lwr** | **upr** | **p adj** |
| --- | --- | --- | --- | --- |
| **Liver-Gill** | 0.001268438 | 9.015145e-05 | 0.002446724 | 0.0359796 |
