# Supplementary material for: Assessing mercury and lead pollution in the Ankobra estuary due to artisanal mining activities: Implications for water quality and aquatic life
Source: PLoS One. 2025 Jun 10;20(6):e0325909. doi: 10.1371/journal.pone.0325909 (PMC12151438; doi:10.1371/journal.pone.0325909)
Supplement: S8 Table — (DOCX) [file pone.0325909.s008.docx]

**S8 Table:** Correlation between fish morphometrics and metal concentrations

| **Size Metric** | **Metal** | **Organ** | **Pearson r** | **p-value** |
| --- | --- | --- | --- | --- |
| Total Length | Hg | Gill | 0.86 | 0.140 |
| Total Length | Hg | Liver | 0.38 | 0.622 |
| Total Length | Pb | Gill | 0.08 | 0.915 |
| Total Length | Pb | Liver | 0.91 | 0.087 |
| Body Weight | Hg | Gill | 0.71 | 0.290 |
| Body Weight | Hg | Liver | 0.30 | 0.703 |
| Body Weight | Pb | Gill | 0.51 | 0.485 |
| Body Weight | Pb | Liver | 0.75 | 0.252 |
