# Supplementary material for: Assessing mercury and lead pollution in the Ankobra estuary due to artisanal mining activities: Implications for water quality and aquatic life
Source: PLoS One. 2025 Jun 10;20(6):e0325909. doi: 10.1371/journal.pone.0325909 (PMC12151438; doi:10.1371/journal.pone.0325909)
Supplement: S9 Table — (DOCX) [file pone.0325909.s009.docx]

**S9 Table:** PCA loadings of physicochemical parameters and toxic metal concentrations on the first two principal components (PC1 and PC2) for the Ankobra estuary.

| **Variable** | **PC1 Loading** | **PC2 Loading** |
| --- | --- | --- |
| Salinity | -0.2418 | -0.4941 |
| DO | -0.3012 | 0.1837 |
| pH | 0.2940 | -0.3604 |
| Turbidity | 0.3264 | 0.4097 |
| Depth | 0.3981 | 0.2393 |
| Hg_w | 0.4870 | 0.0606 |
| Pb_w | 0.3130 | 0.0586 |
| Hg_s | -0.1343 | 0.3359 |
| Pb_s | 0.2240 | -0.2797 |
| Hg_f | -0.3071 | 0.4086 |
| Pb_f | 0.0404 | -0.0442 |
